# Supplementary material for: Conformational Sampling Deciphers the Chameleonic Properties of a VHL-Based Degrader
Source: Pharmaceutics. 2023 Jan 12;15(1):272. doi: 10.3390/pharmaceutics15010272 (PMC9861353; doi:10.3390/pharmaceutics15010272)
Supplement: Supplementary file 1 [file pharmaceutics-15-00272-s001.zip › pharmaceutics-2139513 - supplementary.pdf]

pharmaceutics

MDPI

Supplementary Materials

Conformational Sampling Deciphers the Chameleonic Properties of a VHL-Based Degrader

Giuseppe Ermondi [1], Diego Garcia Jimenez [1], Matteo Rossi Sebastiano [1], Jan Kihlberg [2] and Giulia Caron [1,*]

[1] Department of Molecular Biotechnology and Health Sciences, University of Torino, Quarello 15, 10135 Torino, Italy
[2] Department of Chemistry—BMC, Uppsala University, SE-75123 Uppsala, Sweden
* Correspondence: giulia.caron@unito.it; Tel.: +39-0116708337

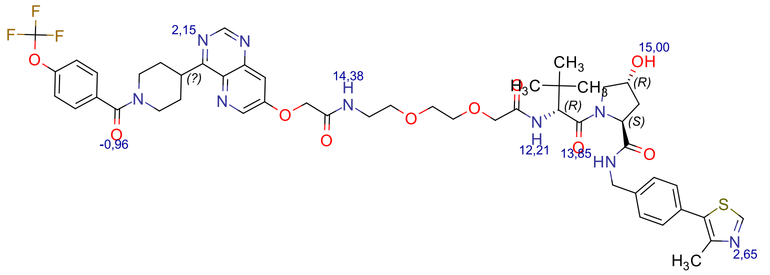

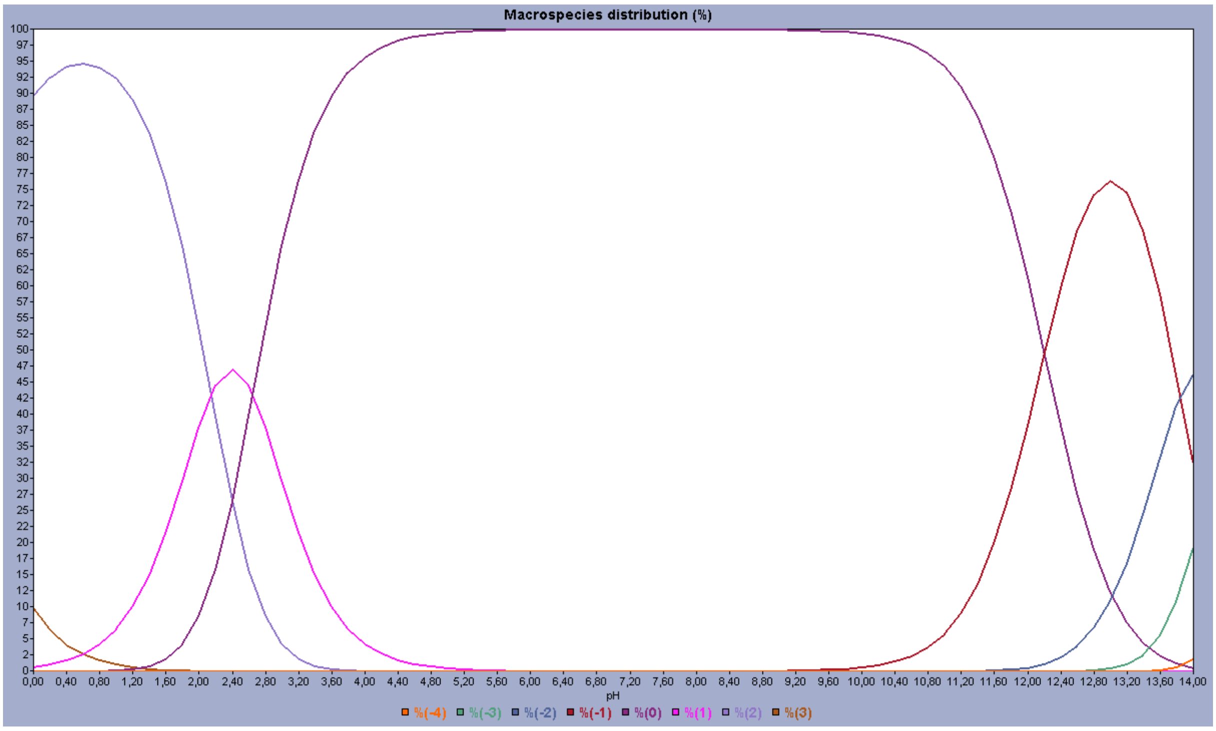

Figure S1. Predicted ionization profile and pKa values of PROTAC-1. ChemAxon tool was used.

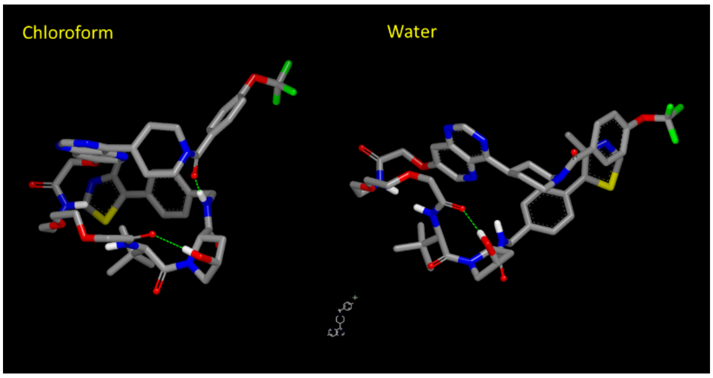

Figure S2. Comparison of the IMHBs pattern in the two solvents from Sc05.

Table S1. 2D descriptors calculated for PROTAC-1. In red Ro5 violations.

MW	RBN	PHI	nHDon	nHAcc	TPSA(Tot)
1034.12	21	19.04	4	22	264.87

Table S2. Summary of the data resulting from the conformational sampling protocols used.

	Sc01		Sc02		Sc03		Sc04		Sc05		Sc06	
	water	$CHCl_3$	water	$CHCl_3$	water	$CHCl_3$	water	$CHCl_3$	water	$CHCl_3$	water	$CHCl_3$
nConf	181	99	275	90	275	90	114	123	331	174	331	174
Rgyr												
Min	5.25	5.23	5.17	5.33	5.17	5.33	5.27	5.29	5.15	5.17	5.15	5.17
Max	6.69	7.01	6.84	6.3	6.84	6.3	6.44	6.99	6.83	6.35	6.83	6.35
Mean	5.78	5.79	5.79	5.96	5.79	5.96	5.73	6.13	5.76	5.6	5.76	5.6
Median	5.76	5.82	5.61	5.98	5.61	5.98	5.6	6.19	5.62	5.51	5.62	5.51
StdDev	0.23	0.26	0.46	0.16	0.46	0.16	0.36	0.36	0.4	0.27	0.4	0.27
Δ (Max water - Min $CHCl_3$)	1.46		1.51		1.51		1.15		1.66		1.66	
3D PSA												
Min	238.7	224.7	238	223.2	238	223.2	241.2	233.4	235.2	230.4	235.2	230.4
Max	258.7	242.6	254.6	237	254.6	237	254.7	250.8	257	250.7	257	250.7
Mean	249.3	235.1	248.1	227.8	248.1	227.8	249.6	240.8	246.2	236.2	246.2	236.2
Median	248.9	235.1	248	227.1	248	227.06	249.82	239.74	247.14	236.03	247.14	236
StdDev	3.41	3.68	2.91	3.08	2.91	3.08	2.44	3.7	4.53	3.09	4.53	3.09
Δ (Max water - Min $CHCl_3$)	34		31.4		31.4		21.3		26.6		26.6	
IMHBs												
Min	0	1	0	2	0	2	0	0	0	1	0	1
Max	3	4	3	4	3	4	2	2	2	2	2	2
Mean	0.97	2.44	1.05	3.13	1.05	3.13	0.53	1.2	1.11	1.93	1.11	1.93
Median	1	2	1	3	1	3	1	1	1	2	1	2
StDev	0.65	0.67	0.48	0.56	0.48	0.56	0.53	0.47	0.54	0.26	0.54	0.26
Δ (Max $CHCl_3$ - Min water)	4		4		4		2		2		2	

Table S3. Summary of the data resulting from the post minimization performed with MOPAC.

MO01		
Starting ensemble	Sc01	
Solvent	water	$CHCl_3$
nConf	100	80
Rgyr		
Min	5.03	5.14
Max	5.9	6.73
⚜	0.87	1.59
Mean	5.37	6.06
Median	5.3	6.03
StdDev	0.22	0.27
Δ (Max water - Min $CHCl_3$)	0.76	

**3D PSA**		
**Min**	220.8	207.9
**Max**	241	232.3
⚜	20.22	24.42
**Mean**	231.2	221.4
**Median**	230.8	221.4
**StdDev**	4.6	5.8
**Δ (Max water - Min $CHCl_3$)**	33.1	
**IMHBs**		
**Min**	1	1
**Max**	3	3
**Mean**	1.6	2.28
**Median**	2	2
**StdDev**	0.62	0.72
**Δ (Max $CHCl_3$ - Min water)**	2	
